# Potential Mechanism of Detoxification of Cyanide Compounds by Gut Microbiomes of Bamboo-Eating Pandas

**DOI:** 10.1128/mSphere.00229-18

**Published:** 2018-06-13

**Authors:** Lifeng Zhu, Zhisong Yang, Ran Yao, Liangliang Xu, Hua Chen, Xiaodong Gu, Tonggui Wu, Xuyu Yang

**Affiliations:** a College of Life Sciences, Nanjing Normal University, Nanjing, China; b University of Nebraska at Omaha, Omaha, Nebraska, USA; c Key Laboratory of Southwest China Wildlife Resources Conservation (Ministry of Education), China West Normal University, Nanchong, China; d Shanghai Biozeron Bioinformatics Center, Shanghai, China; e Sichuan Station for Wildlife Survey and Management, Chengdu, China; f East China Coastal Forest Ecosystem Long-Term Research Station, Research Institute of Subtropical Forestry, Chinese Academy of Forestry, Fuyang, Zhejiang, China; University of Wisconsin-Madison

**Keywords:** bamboo, comparative genomics, cyanide compound detoxification, gut microbial metagenomes, pandas

## Abstract

Gut microbes can enhance the ability of hosts to consume secondary plant compounds and, therefore, expand the dietary niche breadth of mammalian herbivores. The giant and red pandas are bamboo-eating specialists within the mammalian order Carnivora. Bamboo contains abundant plant secondary metabolites (e.g., cyanide-containing compounds). However, Carnivora species, including the giant panda, have deficient levels of rhodanese (one of the essential cyanide detoxification enzymes) in their tissues compared with the same tissues of herbivores. Here, we make a comparative analysis of 94 gut metagenomes, including 25 from bamboo-eating pandas (19 from giant pandas and 6 from red pandas), 30 from Père David’s deer, and 39 from published data for other mammals. The bamboo-eating pandas’ gut microbiomes had some common features, such as high proportions of *Pseudomonas* bacteria. The results revealed that bamboo-eating pandas’ gut microbiomes were significantly enriched in putative genes coding for enzymes related to cyanide degradation (e.g., rhodanese) compared with the gut microbiomes of typical herbivorous mammals, which might have coevolved with their special bamboo diets. The enrichment of putative cyanide-digesting gut microbes, in combination with adaptations related to morphology (e.g., pseudothumbs) and genomic signatures, show that the giant panda and red panda have evolved some common traits to adapt to their bamboo diet.

**IMPORTANCE** The giant panda (Ailuropoda melanoleuca) and red panda (Ailurus fulgens), two obligate bamboo feeders, have distinct phylogenetic positions in the order Carnivora. Bamboo is extraordinarily rich in plant secondary metabolites, such as allied phenolic and polyphenolic compounds and even toxic cyanide compounds. Here, the enrichment of putative cyanide-digesting gut microbes, in combination with adaptations related to morphology (e.g., pseudothumbs) and genomic signatures, show that the giant panda and red panda have evolved some common traits to adapt to their bamboo diet. Thus, here is another story of diet-driven gut microbiota in nature.

## Abstract (precis)

The giant panda (Ailuropoda melanoleuca) and red panda (Ailurus fulgens), two obligate bamboo feeders, have distinct phylogenetic positions in the order Carnivora. Bamboo is extraordinarily rich in plant secondary metabolites, such as allied phenolic and polyphenolic compounds and even toxic cyanide compounds. Here, the enrichment of putative cyanide-digesting gut microbes, in combination with adaptations related to morphology (e.g., pseudothumbs) and genomic signatures, show that the giant panda and red panda have evolved some common traits to adapt to their bamboo diet. Thus, here is another story of diet-driven gut microbiota in nature.

## INTRODUCTION

The foraging ecology of mammalian herbivores is shaped by secondary plant compounds that defend plants against herbivory. However, gut microbes can enhance the ability of hosts to consume secondary plant compounds (1). Therefore, the gut microbes expand the dietary niche breadth of mammalian herbivores (1). Many cyanogenic plants release hydrogen cyanide (HCN) in sufficient quantities to be toxic and, as a result, tend to be avoided by herbivores. However, there are many exceptions, with some herbivores either immune to the cyanogenic status of the plant or, in some cases, attracted to cyanogenic plants (2).

The giant panda (Ailuropoda melanoleuca) and red panda (Ailurus fulgens), two sympatric species, have distinct phylogenetic positions in the order Carnivora (3, 4). The giant panda belongs to the family Ursidae (5), whereas the red panda belongs to the family Ailuridae within the superfamily Musteloidea (6). However, the giant panda and red panda exhibit dietary peculiarities for members of the mammalian order Carnivora by possessing a gastrointestinal tract typical of carnivores yet specializing in bamboo foraging. Giant pandas consume ~12.5 kg per day of highly fibrous bamboo material, including stems, leaves, and shoots (5). Bamboo is extraordinarily rich in plant secondary metabolites, such as allied phenolic and polyphenolic compounds (e.g., tannins), terpenoids, and even toxic cyanide compounds (5, 7–9). Cyanide (a.k.a. hydrogen cyanide, prussic acid, or bitter almond) is a small molecule composed of a carbon and a nitrogen atom joined by a stable triple bond and is a potent metabolic poison of animals (10). Recently, research on captive giant pandas confirmed the absorption (~65%) of cyanide contained in bamboo/bamboo shoots (Chimonobambusa szechuanensis; about 3.2 ± 0.6 µg/g). The daily cyanide absorption was 0.52 ± 0.08 mg/kg of body mass (mean ± standard deviation) for male giant pandas and 0.56 ± 0.05 mg/kg of body mass for female giant pandas. Approximately 80% of the absorbed cyanide was metabolized to less toxic thiocyanate. The authors further found that the levels of host rhodanese expression and activity in liver and kidney of giant pandas were significantly higher than in domestic cats but lower than in herbivorous rabbits (11). The levels of host rhodanese in tissues of animals may reflect the efficacy of the cyanide detoxification function in tissues (12). The findings to date indicate cyanide detoxification by giant pandas but still cannot explain how detoxification occurs (54.8 to 66.1 mg of cyanide daily, close to a fatal dose for a human) by this significantly lower host rhodanese expression and activity compared with those in the herbivorous rabbit.

Host diet and phylogeny both influence gut microbiome communities (13, 14). Some differences in diet were found between the giant panda and red panda. For example, the giant panda consumes both the leaf and stem of bamboo and bamboo shoots (5), whereas the red panda does not eat the stem portion but has a much more variable diet (preferring leaves and shoots) (4, 15, 16). Some differences in gut microbial communities were observed between giant and red pandas (most of them from captive individuals) by using 16S rRNA sequences (17). However, considering their similar diets of bamboo species and bamboo parts (leaves and shoots) in the sympatric wild habitat and their close phylogenetic relatedness, we hypothesized that the sympatric bamboo-eating pandas’ gut microbiomes would commonly be enriched in genes coding for some putative enzymes involved in cyanide detoxification. We tested this hypothesis using gut microbiome metagenomes (functional level) from wild giant pandas, wild red pandas, wild Père David’s deer (Elaphurus davidianus) (as a typical herbivorous mammal used for comparison), and other mammals (published data).

## RESULTS AND DISCUSSION

### Panda diets have a higher relative concentration of cyanide compounds than Père David’s deer diets.

**FIG 1:**
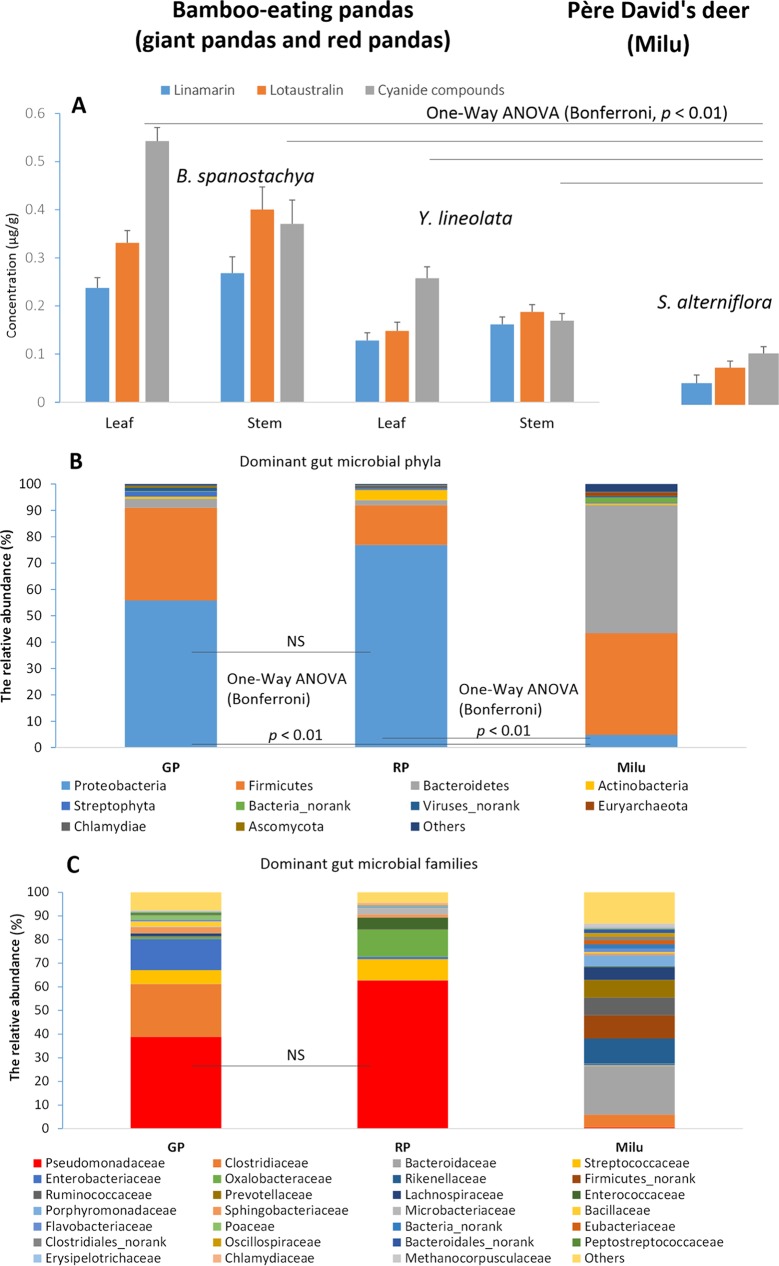
The cyanide compound concentrations in the food of bamboo-eating pandas (giant pandas and red pandas) and Père David’s deer, and the animals’ gut microbial communities. (A) Cyanide compound concentrations in the dietary plants of bamboo-eating pandas and Père David’s deer. (B) Dominant gut microbial phyla in bamboo-eating-panda and Père David’s deer gut microbiomes. (C) Dominant gut microbial families in bamboo-eating-panda and Père David’s deer gut microbiomes. NS, nonsignificant.

In their wild habitats, bamboo-eating pandas can eat over 40 bamboo species, including Bashania spanostachya, Yushania lineolata (major dietary bamboo in the Xiaoxiangling Mountains), and Chimonobambusa szechuanensis (one of the major bamboos in the Daxiangling Mountains) (16, 18). We detected an abundance of cyanogenic glycosides (linamarin and lotaustralin) or total cyanide compounds in the primary food source (bamboo leaves and stems of B. spanostachya and *Y. lineolate*), of pandas (giant pandas and red pandas) from the Xiaoxiangling Mountains (Fig. 1A). However, these cyanide compounds were present in significantly lower concentrations in Spartina alterniflora, the primary food of wild Père David’s deer (Milu) in Dafeng National Natural Reserve (Fig. 1A). For instance, the mean total cyanide compound concentrations were significantly higher in leaves (one-way analysis of variance [ANOVA] between groups, *F* = 466.96, *P* = 0.000; *post hoc* test [Bonferroni], *P* < 0.01) than in stems in either B. spanostachya (0.5425 ± 0.0285 µg/g versus 0.3707 ± 0.0493 µg/g) or Y. lineolata (0.2575 ± 0.0242 µg/g versus 0.1692 ± 0.0151 µg/g). The cyanide concentration of S. alterniflora was significantly lower (0.1040 ± 0.0135 µg/g) than those of bamboos eaten by pandas (one-way ANOVA [Bonferroni], *P* < 0.01) (Fig. 1A).

The Joint FAO/WHO Expert Committee on Food Additives points out that an acute reference dose is 0.09 mg/kg of body weight for cyanide compounds in food, and the derived provisional maximum tolerable daily intake is 0.02 mg/kg of body weight (19). Assuming a wild adult giant panda’s (100 kg) cyanide sensitivity is proportional to that of humans, an average acute reference dose of 9 mg would be found in about 17 (leaves) to 24 (stems) kg of B. spanostachya or 35 (leaves) to 53 (stems) kg of Y. lineolata. The food intake of adult giant pandas has been estimated as 10 to 18 (mean = 12.5) kg of bamboo per day when they forage on leaves and stems and 38 kg when they subsist on bamboo shoots (5). The putative daily intake of cyanide compounds in giant pandas is close to the acute reference dose and higher than the tolerable daily dose for humans. An adult red panda is about 5 kg, and the daily bamboo intake is over 1.5 kg of fresh leaves and 4 kg of new shoots (16). A putative average acute reference dose of 0.45 mg would be estimated to be present in about 0.8 (leaves) to 1.2 (stems) kg of B. spanostachya or 1.7 (leaves) to 2.7 (stems) kg of Y. lineolata. Therefore, both bamboo-eating pandas would be under relatively high cyanide toxin pressure.

### Bamboo-eating pandas (giant pandas and red pandas) have some common dominant gut microbes.

*Proteobacteria* was one of the common dominant phyla in the gut microbial communities, based on taxonomic classifications of predicted gene sequences, with relative abundances of 0.56 ± 0.41 in giant pandas and 0.77 ± 0.34 in red pandas (Fig. 1B). The mean abundance of *Proteobacteria* was significantly higher (one-way ANOVA between groups, *F* = 28.05, *P* = 0.000; *post hoc* test [Bonferroni], *P* < 0.01) in pandas than in Père David’s deer. Another of the common dominant families in the gut microbial communities, based on taxonomic classifications of predicted gene sequences, was *Pseudomonadaceae*, with relative abundances of 0.39 ± 0.40 in giant pandas and 0.63 ± 0.38 in red pandas (Fig. 1C). The mean abundance of *Pseudomonadaceae* was significantly higher (one-way ANOVA between groups, *F* = 19.26, *P* = 0.000; *post hoc* test [Bonferroni] for giant panda versus deer, *P* < 0.01, and for red panda versus deer, *P* < 0.01) in pandas than in Père David’s deer, and the difference between the giant panda and red panda was nonsignificant.

**FIG 2:**
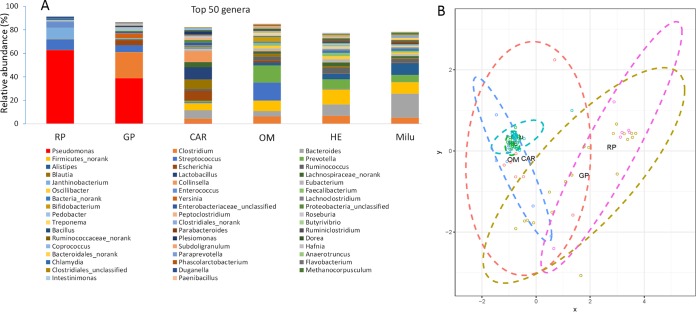
Taxonomic classifications of total predicted gene sequences among 94 mammal gut metagenomes from the following groups: RP, red pandas; GP, giant pandas; CAR, carnivorous mammals; OM, omnivorous mammals; HE, herbivorous mammals; Milu, Père David’s deer. (A) The top 50 genera among the six groups. (B) Nonmetric multidimensional scaling (NMDS) analysis in Vegan using Bray-Curtis distances of putative genus abundances of 94 fecal microbiota revealed potential dissimilar clusters. GP, 19 fresh samples, three of them from Qinling populations (22); CAR (20); OM (20); HE (20).

Comparison to 39 previously published metagenomes (20), including 7 from carnivores (CAR), 11 from omnivores (OM), and 21 from traditional herbivores (HE), confirmed the above-described findings (Fig. 2A). Wild red panda and giant panda gut microbiomes commonly harbored high proportions of *Pseudomonas* bacteria, but the genus was rare in gut microbiomes of other mammals (e.g., other Carnivora species) (Kruskal-Wallis test, *P* < 0.001) (Fig. 2A). Compared to the results of this study and others, including studies of captive giant pandas (13, 21–25), wild and captive red pandas (13, 17, 22), and other herbivorous mammals (13), the mean proportions of *Pseudomonadaceae* were high in red panda populations, particularly in wild red pandas (22). For example, both the giant panda and red panda belong to the phylum Carnivora, but they belong to different phylogenetic families (6). The black bear (Ursus americanus) and spectacled bear (Tremarctos ornatus) are relatives of the giant panda, and they all belong to the family Ursidae. However, the abundances of *Pseudomonas* bacteria in the black bear and spectacled bear gut microbiomes were only 0.13% and 0.06%, respectively. The brown bear (Ursus arctos) gut microbiomes also harbored low abundances of *Pseudomonas* bacteria (6). The relative abundance of *Clostridium* bacteria was highest in giant panda gut microbiomes (Fig. 2A), which has been revealed by several kinds of research (21, 22, 25). A nonmetric multidimensional scaling (NMDS) plot using Bray-Curtis distances of the putative genus abundances of the fecal microbiota showed some dissimilarity between fecal samples of bamboo-eating pandas (giant pandas and red pandas) and those of other mammals, and Père David’s deer (Milu) samples and herbivore (HE) samples formed another cluster (Fig. 2B). Permutational multivariate analysis of variance (PERMANOVA; with 999 permutations) showed that groups of samples were significantly different based on diet (*P* = 0.001), which might reflect some potential correlation between the mammals’ diets and their symbiotic gut microbiomes.

### Bamboo-eating pandas’ gut microbiomes are highly enriched in putative genes coding for enzymes involved in cyanide compound detoxification.

**FIG 3:**
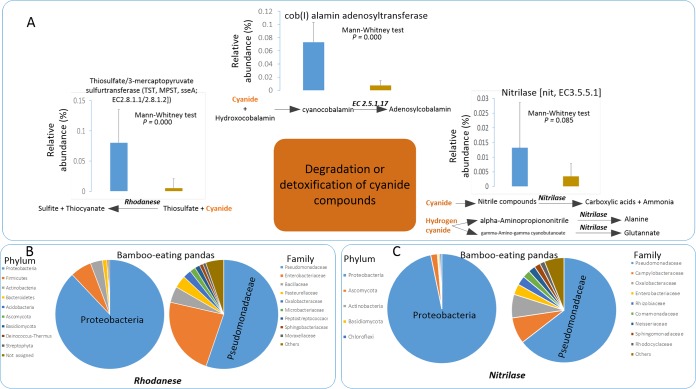
Potential for cyanide compound degradation and detoxification by gut microbes from bamboo-eating pandas (giant pandas and red pandas). (A) The proportions of genes coding for the following putative vital enzymes that are related to the potential degradation and detoxification of cyanide compounds in gut microbiomes of bamboo-eating pandas: nitrilase (Nit) (EC 3.5.5.1), thiosulfate/3-mercaptopyruvate sulfurtransferase (encoded by TST gene, MPST gene, and *sseA*) (EC 2.8.1.1/2.8.1.2), and cob(I)alamin adenosyltransferase (EC 2.5.1.17). Blue bars, bamboo-eating pandas; dark yellow bars, Père David’s deer. (B) The taxonomic assignments of the identified genes (*glpE*, TST gene, MPST gene, and *sseA*) coding for thiosulfate/3-mercaptopyruvate sulfurtransferases (EC 2.8.1.1/2.8.1.2). (C) The taxonomic assignments of the recognized genes coding for nitrilase (EC 3.5.5.1). The relative abundances in panels B and C are the proportions of reads assigned to specific taxa in comparison to total reads (*glpE*, TST gene, MPST gene, and *sseA*) coding for putative thiosulfate/3-mercaptopyruvate sulfurtransferases (EC 2.8.1.1/2.8.1.2) or nitrilase (EC 3.5.5.1).

**FIG 4:**
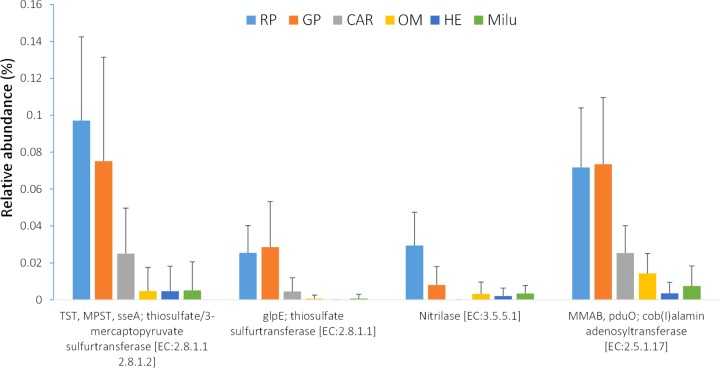
The abundances of genes coding for four putative enzymes involved in cyanide compound degradation and detoxification among 94 gut metagenomes from the following mammal groups: RP, red pandas; GP, giant pandas (19 fresh samples, 3 of them from Qinling populations [22]); CAR, carnivorous mammals (20); OM, omnivorous mammals (20); HE, herbivorous mammals (20); Milu, Père David’s deer.

Cyanide is detoxified by the host enzyme rhodanese (e.g., thiosulfate sulfurtransferase), which is excreted in the urine. We thus investigated the presence of genes coding for putative cyanide-detoxifying sulfur metabolism enzymes in the gut microbiota. The proportion of genes (*glpE*, TST gene, MPST gene, and *sseA*) coding for putative thiosulfate sulfurtransferase (TST)/3-mercaptopyruvate sulfurtransferase (MPST) enzymes (EC 2.8.1.1/2.8.1.2) was significantly higher (Mann-Whitney test, *Z* = −5.43. *P* = 0.000) in panda gut microbiomes than in Père David’s deer gut microbiomes (Fig. 3A; Fig. S1 in the supplemental material). Taxonomic assignment of the genes from the panda microbiomes coding for these enzymes indicated that most of them came from the *Pseudomonas* genus (family *Pseudomonadaceae*) (Fig. 3B; Fig. S2). Nitrilase (Nit) (EC 3.5.5.1) catalyzes the hydrolysis of cyanide to carboxylic acids and ammonia. The proportions of genes coding for this putative enzyme were higher in bamboo-eating-panda gut microbiota (Mann-Whitney test, *Z* = −1.72. *P* = 0.085) than in Père David’s deer gut microbiota, and most of the genes coding for this putative enzyme were assigned to *Pseudomonadaceae* (Fig. 3C). *Post hoc* multiple comparisons among fecal samples from the three groups (giant panda, red panda, and deer) showed that the proportion of genes coding for these putative enzymes was significantly higher in giant pandas or red pandas than in Père David’s deer (one-way ANOVA between groups, *F* = 20.00, *P* = 0.000; *post hoc* test [Bonferroni], giant panda versus deer, *P* < 0.01; red panda versus deer, *P* < 0.01) and that the difference in proportions of these genes between the giant panda and red panda was nonsignificant. Cyanide is also detoxified in combination with hydroxycobalamin (vitamin B_12_) to form cyanocobalamin, which is then excreted in urine and bile. The proportion of genes coding for one putative enzyme [cob(I)alamin adenosyltransferase (2.5.1.17)] that is involved in the last step of coenzyme B_12_ synthesis from cyanocobalamine was significantly higher in panda gut communities (Mann-Whitney test, *Z* = −5.83; *P* = 0.000) than in those from the deer gut microbiomes (Fig. 2A). Both red panda and giant panda gut microbiomes are enriched with genes coding for these putative enzymes involved in cyanide detoxification (Fig. 4; Fig. S3).

10.1128/mSphere.00229-18.1 FIG S1  The proportions (%) of genes coding for the putative *glpE* enzymes (thiosulfate sulfurtransferase [EC 2.8.1.1]) that are related to the potential degradation and detoxification of cyanide compounds in gut microbial communities from 94 mammals. GPRP, bamboo-eating pandas (giant pandas [three of them from Qinling populations] and red pandas); CAR, carnivorous mammals; OM, omnivorous mammals; HE, herbivorous mammals; Milu, Père David’s deer. Download FIG S1, DOCX file, 0.1 MB. Copyright © 2018 Zhu et al. 2018 Zhu et al. This content is distributed under the terms of the Creative Commons Attribution 4.0 International license.

10.1128/mSphere.00229-18.2 FIG S2  The taxonomic classifications (genus level [%]) of predicted gene sequences (*glpE*, TST gene, MPST gene, and *sseA*) coding for putative thiosulfate/3-mercaptopyruvate sulfurtransferases (EC 2.8.1.1/2.8.1.2). Download FIG S2, DOCX file, 0.01 MB. Copyright © 2018 Zhu et al. 2018 Zhu et al. This content is distributed under the terms of the Creative Commons Attribution 4.0 International license.

10.1128/mSphere.00229-18.3 FIG S3  The proportions (%) of genes coding for putative vital enzymes [nitrilase (Nit) (EC 3.5.5.1), thiosulfate/3-mercaptopyruvate sulfurtransferase (EC 2.8.1.1/2.8.1.2), and cob(I)alamin adenosyltransferase (EC 2.5.1.17)] that are related to the potential degradation and detoxification of cyanide compounds in gut microbial communities from 94 mammals. GPRP, bamboo-eating pandas (giant pandas and red pandas); CAR, carnivorous mammals; OM, omnivorous mammals; HE, herbivorous mammals; Deer, Père David’s deer in this study. The ANOVA test on each putative enzyme was significant (*P* < 0.001) in all cases. Download FIG S3, DOCX file, 0.02 MB. Copyright © 2018 Zhu et al. 2018 Zhu et al. This content is distributed under the terms of the Creative Commons Attribution 4.0 International license.

Many strains from the *Pseudomonas* genus play essential roles in the detoxification and degradation of cyanide compounds in the environment (26–31). For instance, one of the most abundant *Pseudomonas* species in bamboo-eating pandas was putatively assigned as Pseudomonas fluorescens (6%). Some studies have found that Pseudomonas fluorescens is involved in cyanide utilization and degradation (28, 32). Thus, we speculated that bamboo-eating-panda gut microbiomes might play a similar role in cyanide compound detoxification and mitigate the effects arising from the ingestion of large concentrations of these compounds in the bamboo diet.

### The coding genes involved in cyanide compound detoxification in 267 published bacterial genomes.

Considering that most of the above-described putative genes came from five families (*Pseudomonadaceae*, *Enterobacteriaceae*, *Comamonadaceae*, *Oxalobacteraceae*, and *Clostridiaceae*), we reannotated 267 published whole bacterial genomes from these five families to confirm the existence of these coding genes in these families (Table S2). Based on 267 whole bacterial genomes, there was enrichment of genes putatively coding for thiosulfate sulfurtransferases (EC 2.8.1.1) in members of both the *Pseudomonadaceae* (36 of 41 genomes) and *Enterobacteriaceae* (70 of 81 genomes), which were the dominant microbial taxa in panda gut microbiomes (Table S3). However, the genes coding for putative thiosulfate sulfurtransferases in genomes from the *Comamonadaceae*, *Oxalobacteraceae*, and *Clostridiaceae* families were not detected in this study (Table S3). Moreover, the abundance of genomes containing genes coding for putative thiosulfate/3-mercaptopyruvate sulfurtransferases was highest in *Pseudomonadaceae* (36 of a total of 41 genomes [88%]) and lowest in *Clostridiaceae* (2 of 88 whole genomes [2%]) (Table S3). Thus, the high enzymatic capacity for putative cyanide compound degradation in these *Pseudomonadaceae* genomes helps in understanding the diet-driven gut microbiomes in bamboo-eating pandas.

### Conclusions.

The giant panda and red panda are obligate bamboo feeders that evolved independently from carnivorous ancestors and possess a convergent phenotype (e.g., pseudothumbs). The pseudothumb, an enlarged radial sesamoid, has been reported to function as an active manipulator, enabling pandas to grasp bamboo stems between the bone and the opposing palm (33–36). Comparative genomic analyses reveal adaptively convergent genes potentially involved with pseudothumb development (limb development genes *DYNC2H1* and *PCNT*) and essential bamboo nutrient utilization (37). Here, bamboo-eating-panda gut microbiomes also harbored some common features (e.g., high proportions of *Pseudomonas* bacteria) and, compared with the levels in microbiomes of other mammals, were enriched with putative genes coding for some enzymes involved in potential degradation or detoxification of cyanide compounds, and these features might be coevolved with their particular bamboo diet.

Moreover, seasonal variations in the hydrogen cyanide concentrations of some plants have been shown (38, 39), and giant pandas undergo seasonal variations in both dietary behavior (e.g., seasonal shifts in plant part consumption) (5, 40–42) and gut microbiome communities (21, 24, 25, 43). For example, two captive giant pandas altered their bamboo consumption behaviors, showing sharply decreased leaf preference in April 2010 and returning to high levels of leaf preference from June to October, corresponding to significant shifts in the densities of total aerobes, streptococci, lactobacilli, and *Bacteroides* spp. (24). Wild giant pandas use different parts of bamboo (shoots, leaves, and stems) and different bamboo species at different times of the year (5). During the period of leaf consumption, bacterial species with genes involved in raw fiber utilization and cell cycle control were overrepresented based on fresh leaf diet-type feces in Qinling Mountains, which might be related to variation in the nutritional quality of bamboo parts. Seasonal nutritional quality variation in wild giant pandas substantially influences gut microbiome composition and function. In this study, variation in cyanide compound concentrations with respect to bamboo species and parts was detected in the same season in Xiaoxiangliang Mountains. The abundance of some of these genes coding for putative cyanide compound degradation enzymes in leaf diet-type feces was higher than in stem diet-type feces, but the difference was nonsignificant (Fig. S4). Although the seasonal changes in cyanide compound concentrations of these dietary bamboos are unknown, these variations in bamboo cyanide compounds and panda dietary behavior may also play a potential role in exposure and subsequent detoxification. Studies integrating plant herbivory defense, panda dietary behavior, and gut microbiomes in wild mountain pandas at population scale will be necessary.

10.1128/mSphere.00229-18.4 FIG S4  The potential for cyanide compound degradation and detoxification by gut microbes in leaf diet-type and stem diet-type fecal samples from giant pandas. (A) The proportions of genes (TST gene, MPST gene, and *sseA*) coding for the putative vital enzymes thiosulfate/3-mercaptopyruvate sulfurtransferase (EC 2.8.1.1/2.8.1.2). (B) The proportions of genes coding for putative GlpE (thiosulfate sulfurtransferase) (EC 2.8.1.1). (C) The proportions of genes coding for putative nitrilase (Nit) (EC 3.5.5.1). (D) The proportions of genes coding for putative cob(I)alamin adenosyltransferase (EC 2.5.1.17). Download FIG S4, DOCX file, 1.2 MB. Copyright © 2018 Zhu et al. 2018 Zhu et al. This content is distributed under the terms of the Creative Commons Attribution 4.0 International license.

Gut microbes of animal herbivores may facilitate the ingestion of toxic plants (1, 44–46). For example, a pilot study reveals that gut microbes are crucial in allowing woodrats (Neotoma lepida) to consume the highly toxic creosote bush (Larrea tridentata). Creosote toxins altered the population structure of the gut microbiome to facilitate an increase in the abundance of genes that metabolize toxic compounds. The importance of this pioneering research is in showing that woodrats are unable to consume creosote toxins after the microbiota is disrupted with antibiotics (1). Therefore, implementing fecal microbiota transplants from giant and red pandas into germ-free mice will help to further quantify the contribution to detoxification by gut microbiomes compared to the detoxification by the hosts themselves.

## MATERIALS AND METHODS

### Sample collection and DNA extraction.

Fresh fecal samples from giant pandas and red pandas in the Xiaoxiangling Mountains were collected from 2012 to 2016, including samples from four giant pandas translocated to this mountain (Luxin [LX], Zhangxiang [ZX], Taotao [TT], and Huajiao [HJ]). Fecal samples from these translocated pandas were collected directly from the GPS-collared individuals by a monitoring team. Fresh fecal samples were frozen upon collection and then shipped on dry ice to the laboratory for analysis. A total of 16 fresh fecal samples from giant pandas and 6 fresh fecal samples from red pandas were used for metagenomic sequencing. Fresh samples of bamboo foraged by pandas (leaves and stems from Bashania spanostachya and Yushania lineolata) were also collected in the Xiaoxiangling Mountains. All samples were frozen upon collection and shipped on dry ice to the laboratory for analysis, as described above (Table S1).

10.1128/mSphere.00229-18.5 TABLE S1  The detailed gut metagenomic information for bamboo-eating pandas and Père David’s deer. Download TABLE S1, DOCX file, 0.02 MB. Copyright © 2018 Zhu et al. 2018 Zhu et al. This content is distributed under the terms of the Creative Commons Attribution 4.0 International license.

10.1128/mSphere.00229-18.6 TABLE S2  The 267 published whole bacterial genomes used for reannotation. Download TABLE S2, DOCX file, 0.02 MB. Copyright © 2018 Zhu et al. 2018 Zhu et al. This content is distributed under the terms of the Creative Commons Attribution 4.0 International license.

10.1128/mSphere.00229-18.7 TABLE S3  The numbers of the enzymes involved in cyanide detoxification based on our reannotation of these 267 published whole bacterial genomes. Download TABLE S3, DOCX file, 0.01 MB. Copyright © 2018 Zhu et al. 2018 Zhu et al. This content is distributed under the terms of the Creative Commons Attribution 4.0 International license.

Fresh fecal samples from Père David’s deer (Elaphurus davidianus [Milu]) were collected during field monitoring from 2014. Fresh fecal samples (*n* = 24) were collected from the Dafeng Père David’s Deer National Preserve over this period, and 6 samples were collected from the Shishou Père David’s Deer National Preserve in November 2014 for metagenomics sequencing. All deer samples were frozen and shipped upon collection as described above (Table S1). Smooth cordgrass (Spartina alterniflora), the primary food of deer at the Dafeng Père David’s Deer National Preserve, was also collected and handled as described above, in November 2016.

Total DNA was extracted from fecal samples using Qiagen DNA stool kits (Qiagen, Germany) according to the manufacturer’s protocols.

### Metagenomic sequencing of microbial communities from bamboo-eating pandas and Milu.

Metagenomic sequencing (including community DNA from 16 giant panda fecal samples, 6 red panda fecal samples, and 30 Milu fecal samples) (Table S1) was performed by Shanghai Biozeron Biotechnology Co., Ltd. (Shanghai, China.) A library was constructed with an average insert size of 450 bp for each sample. Sequencing was performed using an Illumina HiSeq 2500 platform. Illumina Genome Analyzer HiSeq reads were filtered using custom Perl scripts and Trimmomatic (47) to remove (i) all reads less than 50 bp in length, (ii) reads with degenerate bases (N’s), and (iii) all duplicates, defined as sequences whose initial 20 nucleotides were identical and whose overall identity was >97% throughout the length of the shortest read. Raw short reads were compared against the host genome to facilitate the removal of host genomic sequences. The resultant clean, high-quality reads were assembled to generate contigs using the SOAPdenovo assembler (48). Taxonomic classifications of predicted gene sequences were determined using MEGAN5 (49). CD-HIT was used to construct nonredundant gene sets with less than 90% overlap and less than 95% shared sequence identity (50). The SOAPdenovo assembler was used to generate a gene profile for each metagenomics sample (48). Based on these gene profiles, nonredundant gene sequences were searched against the KEGG database using BLASTP (51). A sequence read was annotated as the most optimal hit in the database if (i) the *E* value was <10^−5^, (ii) the bit score was >50, and (iii) the alignment was at least 50% identical between the query and subject. If two entries in the database had equivalent BLAST scores and were both deemed best hits, the read was annotated with both entries. The Kyoto Encyclopedia of Genes and Genomes (KEGG), KEGG Orthology (KO), and Enzyme Commission (EC) pathways associated with each sequence were determined and were converted to a QIIME-readable biom format. The taxonomic distribution of metagenomic reads was again determined using MEGAN (49). Nonredundant gene sequences were searched against the NCBI nonredundant protein database using BLASTX. We further incorporated previously published metagenomic data sets (20, 22). The relative abundances of taxa from all data were used in further STAMP analysis (52). NMDS analysis in Vegan using the Bray-Curtis distances of abundances of putative genera of the fecal microbiota was applied to reveal potential dissimilar clusters (53, 54). PERMANOVA (with 999 permutations) was used to test whether groups of samples were significantly different based on diet. PERMANOVA is nonparametric; significance is determined through permutations.

### Concentrations of cyanide-containing compounds in bamboo and smooth cordgrass.

Forty bamboo samples (28 B. spanostachya and 12 Y. lineolata) and 10 cordgrass samples (S. alterniflora) were chopped, and 10.0 g of powder of each sample was then placed in a 250-ml distillation bottle with distilled water and tartaric acid for downstream analysis. Spectrophotometric analysis with isonicotinic acid pyrazolone was used to determine the concentrations of cyanide-containing compounds as described in the GB/T 5009.36-2003 method (commonly used in the study of hygienic standards of grains) (55). Each sample had two replicates. IBM SPSS software was used to conduct one-way ANOVA (*post hoc* multiple comparisons [Bonferroni]) to investigate how different animal species’ diets affected mean cyanide levels. The threshold for significance was a *P* value of <0.01.

### Data availability.

The metagenomic data have been deposited in figshare at https://doi.org/10.6084/m9.figshare.6303713.
