# Supplementary material for: Potential Mechanism of Detoxification of Cyanide Compounds by Gut Microbiomes of Bamboo-Eating Pandas
Source: mSphere. 2018 Jun 13;3(3):e00229-18. doi: 10.1128/mSphere.00229-18 (PMC6001608; doi:10.1128/mSphere.00229-18)
Supplement: TABLE S1 [file sph003182564st1.docx]

| Sample ID | Clean-Reads | Clean-Bases | Average Length | Origin | Collecting time | Species | Composition |
| --- | --- | --- | --- | --- | --- | --- | --- |
| 19 | 67,881,149 | 9,867,464,337 | 145.36 | XXL | 04/14/2015 | Giant panda | Stems |
| 20131124-ZX-F03 | 62,349,047 | 9,151,635,679 | 146.78 | ZX | 11/24/2013 | Giant panda | Leaves |
| 20140128-zx-F03 | 77,774,061 | 11,537,910,631 | 148.35 | ZX | 01/28/2014 | Giant panda | Leaves |
| 23 | 53,538,195 | 7,772,448,526 | 145.18 | XXL | 07/20/2015 | Giant panda | Stems |
| 31 | 53,166,997 | 7,488,933,645 | 140.86 | ZX | 10/26/2014 | Giant panda | Leaves |
| 34 | 53,452,895 | 7,766,232,979 | 145.29 | HJ | 01/21/2016 | Giant panda | Leaves |
| 50 | 53,316,102 | 7,612,446,370 | 142.78 | TT | 05/11/2015 | Giant panda | Stems |
| 9 | 57,047,483 | 8,411,453,987 | 147.45 | HJ | 11/22/2015 | Giant panda | Leaves |
| A4 | 60,752,300 | 8,992,034,059 | 148.01 | RP | 05/20/2013 | Red panda | Shoots |
| B1-2 | 65,181,154 | 9,677,508,178 | 148.47 | RP | 05/10/2013 | Red panda | Leaves |
| B2-4 | 61,454,276 | 9,120,341,274 | 148.41 | RP | 03/28/2014 | Red panda | Leaves |
| C14 | 56,064,271 | 8,299,373,106 | 148.03 | RP | 03/18/2014 | Red panda | Leaves |
| C18 | 56,456,530 | 8,366,617,158 | 148.2 | RP | 03/18/2014 | Red panda | Leaves |
| C2 | 62,648,157 | 9,305,147,273 | 148.53 | RP | 03/18/2014 | Red panda | Leaves |
| CDXM18 | 57,156,434 | 8,444,257,007 | 147.74 | XXL | 04/06/2014 | Giant panda | Stems |
| CDXM27 | 56,479,002 | 8,300,348,076 | 146.96 | XXL | 03/16/2014 | Giant panda | Leaves |
| CDXM39 | 65,886,310 | 9,753,746,424 | 148.04 | XXL | 03/28/2014 | Giant panda | Stems |
| CDXM40 | 80,837,508 | 11,939,579,426 | 147.7 | XXL | 03/28/2014 | Giant panda | Stems |
| LX3 | 23,696,922 | 2,370,762,117 | 100.05 | LX | 05/26/2013 | Giant panda | Shoots |
| LZP1 | 16,688,791 | 1,660,690,975 | 99.51 | XXL | 05/21/2013 | Giant panda | Stems |
| TT1 | 6,559,947 | 634,818,332 | 96.77 | TT | 11/25/2012 | Giant panda | Leaves |
| TT2 | 44,545,940 | 4,407,262,711 | 98.94 | TT | 03/07/2013 | Giant panda | Stems |
| DF2_15 | 51,284,128 | 6,168,691,424 | 120.28 | DF2 | 09/25/2014 | Père David’s deer | |
| DF2_20 | 71,325,102 | 8,582,260,058 | 120.33 | DF2 | 09/25/2014 | Père David’s deer | |
| DF2_24 | 52,060,329 | 6,266,304,988 | 120.37 | DF2 | 09/25/2014 | Père David’s deer | |
| DF2_25 | 50,158,469 | 6,104,959,666 | 121.71 | DF2 | 09/25/2014 | Père David’s deer | |
| DF3_19 | 51,831,871 | 6,264,280,197 | 120.86 | DF3 | 09/26/2014 | Père David’s deer | |
| DF3_23 | 57,763,113 | 7,013,725,486 | 121.42 | DF3 | 09/26/2014 | Père David’s deer | |
| DF3_28 | 45,872,353 | 5,605,033,706 | 122.19 | DF3 | 09/26/2014 | Père David’s deer | |
| DF3_31 | 57,481,821 | 6,981,300,662 | 121.45 | DF3 | 09/26/2014 | Père David’s deer | |
| DFW1_15 | 44,471,287 | 5,339,258,433 | 120.06 | DF1 | 11/26/2014 | Père David’s deer | |
| DFW1_23 | 95,046,468 | 11,649,563,409 | 122.57 | DF1 | 11/26/2014 | Père David’s deer | |
| DFW1_29 | 59,833,093 | 7,257,653,921 | 121.3 | DF1 | 11/26/2014 | Père David’s deer | |
| DFW1_34 | 45,782,105 | 5,512,593,185 | 120.41 | DF1 | 11/26/2014 | Père David’s deer | |
| DFW2_14 | 83,046,788 | 10,003,180,165 | 120.45 | DF2 | 11/26/2014 | Père David’s deer | |
| DFW2_19 | 58,982,533 | 7,151,517,321 | 121.25 | DF2 | 11/26/2014 | Père David’s deer | |
| DFW2_26 | 36,532,749 | 4,435,302,103 | 121.41 | DF2 | 11/26/2014 | Père David’s deer | |
| DFW2_35 | 48,566,364 | 5,890,782,249 | 121.29 | DF2 | 11/26/2014 | Père David’s deer | |
| DFW3_19 | 48,149,754 | 5,836,143,232 | 121.21 | DF3 | 11/27/2014 | Père David’s deer | |
| DFW3_27 | 52,064,152 | 6,304,905,735 | 121.1 | DF3 | 11/27/2014 | Père David’s deer | |
| DFW3_38 | 57,062,169 | 6,823,668,122 | 119.58 | DF3 | 11/27/2014 | Père David’s deer | |
| DFW3_45 | 64,078,282 | 7,691,988,419 | 120.04 | DF3 | 11/27/2014 | Père David’s deer | |
| ED1_13 | 62,339,201 | 7,479,739,750 | 119.98 | DF1 | 09/25/2014 | Père David’s deer | |
| ED1_17 | 84,874,651 | 10,192,740,387 | 120.09 | DF1 | 09/25/2014 | Père David’s deer | |
| ED1_21 | 112,668,144 | 13,597,298,685 | 120.68 | DF1 | 09/25/2014 | Père David’s deer | |
| ED1_24 | 79,405,567 | 9,570,213,275 | 120.52 | DF1 | 09/25/2014 | Père David’s deer | |
| SH_10 | 55,902,784 | 6,822,473,125 | 122.04 | HB | 11/05/2014 | Père David’s deer | |
| SH_16 | 52,529,241 | 6,398,252,196 | 121.8 | HB | 11/05/2014 | Père David’s deer | |
| SH_23 | 45,874,768 | 5,601,678,319 | 122.11 | HB | 11/05/2014 | Père David’s deer | |
| SH_31 | 52,628,151 | 6,433,289,625 | 122.24 | HB | 11/06/2014 | Père David’s deer | |
| SH_45 | 61,260,927 | 7,438,240,913 | 121.42 | HB | 11/06/2014 | Père David’s deer | |
| SH_60 | 73,647,157 | 8,904,561,252 | 120.91 | HB | 11/06/2014 | Père David’s deer |  |

XXL, Xiaoxiangling Mountain population. LX, Luxin, a name of a translocated giant panda individual; ZX, Zhangxiang, a name of a translocated individual; TT, Taotao, a name of a translocated individual; HJ, Huajiao, a name of a translocated individual.

DF1, core area 1 in Jiangsu Dafeng Père David’s deer National Natural Reserve; DF2, core area 2 in Jiangsu Dafeng National Natural Reserve; DF3, core area 3 in Jiangsu Dafeng National Natural Reserve; HB, Hubei Shishou Père David’s deer National Natural Reserve. Composition, mean the dominant composition in bamboo-eating panda’s fresh feces.
