# Supplementary material for: Potential Mechanism of Detoxification of Cyanide Compounds by Gut Microbiomes of Bamboo-Eating Pandas
Source: mSphere. 2018 Jun 13;3(3):e00229-18. doi: 10.1128/mSphere.00229-18 (PMC6001608; doi:10.1128/mSphere.00229-18)
Supplement: TABLE S2 [file sph003182564st2.docx]

| Family | Genus | Species | RefSeq accession |
| --- | --- | --- | --- |
| Clostridiaceae | Acetivibrio | *Acetivibrio cellulolyticus CD2* | GCF_000179595.2 |
| Clostridiaceae | Acetivibrio | *Acetivibrio ethanolgignens* | GCF_001461035.1 |
| Clostridiaceae | Alkaliphilus | *Alkaliphilus metalliredigens QYMF* | GCF_000016985.1 |
| Clostridiaceae | Alkaliphilus | *Alkaliphilus oremlandii OhILAs* | GCF_000018325.1 |
| Clostridiaceae | Alkaliphilus | *Alkaliphilus transvaalensis ATCC 700919* | GCF_000621485.1 |
| Clostridiaceae | Anaerostipes | *Anaerostipes caccae DSM 14662* | GCF_000154305.1 |
| Clostridiaceae | Anaerostipes | *Anaerostipes hadrus DSM 3319* | GCF_000332875.1 |
| Clostridiaceae | Anaerotruncus | *Anaerotruncus colihominis DSM 17241* | GCF_000154565.1 |
| Clostridiaceae | Butyricicoccus | *Butyricicoccus pullicaecorum 1.2* | GCF_000398925.1 |
| Clostridiaceae | Caloramator | *Caloramator australicus RC3* | GCF_000297115.1 |
| Clostridiaceae | Caloramator | *Caloramator mitchellensis* | GCF_001440545.1 |
| Clostridiaceae | Caloranaerobacter | *Caloranaerobacter azorensis H53214* | GCF_000761095.1 |
| Clostridiaceae | Caloranaerobacter | *Caloranaerobacter sp. TR13* | GCF_001316435.1 |
| Clostridiaceae | Clostridium | *Clostridium tetani E88* | GCF_000007625.1 |
| Clostridiaceae | Clostridium | *Clostridium acetobutylicum ATCC 824* | GCF_000008765.1 |
| Clostridiaceae | Clostridium | *Clostridium perfringens str. 13* | GCF_000009685.1 |
| Clostridiaceae | Clostridium | *Clostridium novyi NT* | GCF_000014125.1 |
| Clostridiaceae | Clostridium | *Clostridium kluyveri DSM 555* | GCF_000016505.1 |
| Clostridiaceae | Clostridium | *Clostridium beijerinckii NCIMB 8052* | GCF_000016965.1 |
| Clostridiaceae | Clostridium | *Clostridium botulinum A str. Hall* | GCF_000017045.1 |
| Clostridiaceae | Clostridium | *Clostridium botulinum B str. Eklund 17B (NRP)* | GCF_000020165.1 |
| Clostridiaceae | Clostridium | *Clostridium cellulolyticum H10* | GCF_000022065.1 |
| Clostridiaceae | Clostridium | *Clostridium botulinum A str. ATCC 3502* | GCF_000063585.1 |
| Clostridiaceae | Clostridium | *Clostridium saccharolyticum WM1* | GCF_000144625.1 |
| Clostridiaceae | Clostridium | *Clostridium cellulovorans 743B* | GCF_000145275.1 |
| Clostridiaceae | Clostridium | *Clostridium leptum DSM 753* | GCF_000154345.1 |
| Clostridiaceae | Clostridium | *Clostridium scindens ATCC 35704* | GCF_000154505.1 |
| Clostridiaceae | Clostridium | *Clostridium spiroforme DSM 1552* | GCF_000154805.1 |
| Clostridiaceae | Clostridium | *Clostridium hiranonis DSM 13275* | GCF_000156055.1 |
| Clostridiaceae | Clostridium | *Clostridium hylemonae DSM 15053* | GCF_000156515.1 |
| Clostridiaceae | Clostridium | *Clostridium asparagiforme DSM 15981* | GCF_000158075.1 |
| Clostridiaceae | Clostridium | *Clostridium methylpentosum DSM 5476* | GCF_000158655.1 |
| Clostridiaceae | Clostridium | *Clostridium carboxidivorans P7* | GCF_000163855.1 |
| Clostridiaceae | Clostridium | *Clostridium papyrosolvens DSM 2782* | GCF_000175795.2 |
| Clostridiaceae | Clostridium | *Clostridium lentocellum DSM 5427* | GCF_000178835.2 |
| Clostridiaceae | Clostridium | *Clostridium symbiosum WAL-14163* | GCF_000189595.1 |
| Clostridiaceae | Clostridium | *Clostridium sticklandii* | GCF_000196455.1 |
| Clostridiaceae | Clostridium | *Clostridium citroniae WAL-17108* | GCF_000233455.1 |
| Clostridiaceae | Clostridium | *Clostridium clariflavum DSM 19732* | GCF_000237085.1 |
| Clostridiaceae | Clostridium | *Clostridium arbusti SL206* | GCF_000246895.1 |
| Clostridiaceae | Clostridium | *Clostridium senegalense JC122* | GCF_000285575.1 |
| Clostridiaceae | Clostridium | *Clostridium celatum DSM 1785* | GCF_000320405.1 |
| Clostridiaceae | Clostridium | *Clostridium stercorarium subsp. stercorarium DSM 8532* | GCF_000331995.1 |
| Clostridiaceae | Clostridium | *Clostridium saccharoperbutylacetonicum N1-4(HMT)* | GCF_000340885.1 |
| Clostridiaceae | Clostridium | *Clostridium ultunense Esp* | GCF_000344075.2 |
| Clostridiaceae | Clostridium | *Clostridium termitidis CT1112* | GCF_000350485.1 |
| Clostridiaceae | Clostridium | *Clostridium innocuum 2959* | GCF_000371425.1 |
| Clostridiaceae | Clostridium | *Clostridium colicanis 209318* | GCF_000371465.1 |
| Clostridiaceae | Clostridium | *Clostridium butyricum 60E.3* | GCF_000371625.1 |
| Clostridiaceae | Clostridium | *Clostridium bolteae 90A9* | GCF_000371705.1 |
| Clostridiaceae | Clostridium | *Clostridium sporosphaeroides DSM 1294* | GCF_000383295.1 |
| Clostridiaceae | Clostridium | *Clostridium pasteurianum BC1* | GCF_000389635.1 |
| Clostridiaceae | Clostridium | *Clostridium sartagoforme AAU1* | GCF_000401215.1 |
| Clostridiaceae | Clostridium | *Clostridium paraputrificum AGR2156* | GCF_000424025.1 |
| Clostridiaceae | Clostridium | *Clostridium cadaveris AGR2141* | GCF_000424205.1 |
| Clostridiaceae | Clostridium | *Clostridium tyrobutyricum DSM 2637 = ATCC 25755 = JCM 11008* | GCF_000429805.1 |
| Clostridiaceae | Clostridium | *Clostridium sordellii VPI 9048* | GCF_000444095.1 |
| Clostridiaceae | Clostridium | *Clostridium bifermentans ATCC 638* | GCF_000452245.1 |
| Clostridiaceae | Clostridium | *Clostridium intestinale URNW* | GCF_000469625.1 |
| Clostridiaceae | Clostridium | *Clostridium saccharobutylicum DSM 13864* | GCF_000473995.1 |
| Clostridiaceae | Clostridium | *Clostridium autoethanogenum DSM 10061* | GCF_000484505.1 |
| Clostridiaceae | Clostridium | *Clostridium ultunense DSM 10521* | GCF_000511955.1 |
| Clostridiaceae | Clostridium | *Clostridium josui JCM 17888* | GCF_000526495.1 |
| Clostridiaceae | Clostridium | *Clostridium indolis DSM 755* | GCF_000526995.1 |
| Clostridiaceae | Clostridium | *Clostridium cellulosi CS-4-4* | GCF_000582395.1 |
| Clostridiaceae | Clostridium | *Clostridium lundense DSM 17049* | GCF_000619945.1 |
| Clostridiaceae | Clostridium | *Clostridium viride DSM 6836* | GCF_000620945.1 |
| Clostridiaceae | Clostridium | *Clostridium tetanomorphum DSM 665* | GCF_000647895.1 |
| Clostridiaceae | Clostridium | *Clostridium saccharogumia DSM 17460* | GCF_000686665.1 |
| Clostridiaceae | Clostridium | *Clostridium hydrogeniformans DSM 21757* | GCF_000686705.1 |
| Clostridiaceae | Clostridium | *Clostridium akagii DSM 12554* | GCF_000686725.1 |
| Clostridiaceae | Clostridium | *Clostridium aerotolerans DSM 5434* | GCF_000687555.1 |
| Clostridiaceae | Clostridium | *Clostridium mangenotii LM2* | GCF_000687955.1 |
| Clostridiaceae | Clostridium | *Clostridium litorale DSM 5388* | GCF_000699585.1 |
| Clostridiaceae | Clostridium | *Clostridium algidicarnis* | GCF_000703125.1 |
| Clostridiaceae | Clostridium | *Clostridium aminophilum DSM 10710* | GCF_000711825.1 |
| Clostridiaceae | Clostridium | *Clostridium pasteurianum DSM 525 = ATCC 6013* | GCF_000724205.1 |
| Clostridiaceae | Clostridium | *Clostridium sulfidigenes* | GCF_000732635.1 |
| Clostridiaceae | Clostridium | *Clostridium glycyrrhizinilyticum JCM 13369* | GCF_001311035.1 |
| Clostridiaceae | Clostridium | *Clostridium sp. ND2* | GCF_001403635.1 |
| Clostridiaceae | Clostridium | *Clostridium butyricum* | GCF_001456065.2 |
| Clostridiaceae | Clostridium | *Clostridium neonatale* | GCF_001458595.1 |
| Clostridiaceae | Clostridium | *Clostridium ventriculi* | GCF_001485185.1 |
| Clostridiaceae | Dorea | *Dorea longicatena DSM 13814* | GCF_000154065.1 |
| Clostridiaceae | Dorea | *Dorea formicigenerans ATCC 27755* | GCF_000169235.1 |
| Clostridiaceae | Oxobacter | *Oxobacter pfennigii* | GCF_001317355.1 |
| Clostridiaceae | Subdoligranulum | *Subdoligranulum variabile DSM 15176* | GCF_000157955.1 |
| Clostridiaceae | Thermobrachium | *Thermobrachium celere DSM 8682* | GCF_000430995.1 |
| Comamonadaceae | Acidovorax | *Acidovorax citrulli AAC00-1* | GCF_000015325.1 |
| Comamonadaceae | Acidovorax | *Acidovorax ebreus TPSY* | GCF_000022305.1 |
| Comamonadaceae | Acidovorax | *Acidovorax delafieldii 2AN* | GCF_000175235.1 |
| Comamonadaceae | Acidovorax | *Acidovorax radicis N35* | GCF_000204195.1 |
| Comamonadaceae | Acidovorax | *Acidovorax caeni* | GCF_001298675.1 |
| Comamonadaceae | Acidovorax | *Acidovorax sp. Leaf160* | GCF_001424265.1 |
| Comamonadaceae | Acidovorax | *Acidovorax sp. Root217* | GCF_001428645.1 |
| Comamonadaceae | Alicycliphilus | *Alicycliphilus denitrificans K601* | GCF_000204645.1 |
| Comamonadaceae | Brachymonas | *Brachymonas chironomi DSM 19884* | GCF_000374625.1 |
| Comamonadaceae | Caldimonas | *Caldimonas manganoxidans ATCC BAA-369* | GCF_000381125.1 |
| Comamonadaceae | Comamonas | *Comamonas testosteroni CNB-2* | GCF_000093145.3 |
| Comamonadaceae | Comamonas | *Comamonas composti DSM 21721* | GCF_000429845.1 |
| Comamonadaceae | Comamonas | *Comamonas badia DSM 17552* | GCF_000484635.1 |
| Comamonadaceae | Comamonas | *Comamonas aquatica NBRC 14918* | GCF_000739875.1 |
| Comamonadaceae | Comamonas | *Comamonas granuli NBRC 101663* | GCF_000739995.1 |
| Comamonadaceae | Comamonas | *Comamonas kerstersii* | GCF_001294445.1 |
| Comamonadaceae | Curvibacter | *Curvibacter lanceolatus ATCC 14669* | GCF_000381265.1 |
| Comamonadaceae | Curvibacter | *Curvibacter sp. PAE-UM* | GCF_001432305.1 |
| Comamonadaceae | Delftia | *Delftia acidovorans SPH-1* | GCF_000018665.1 |
| Comamonadaceae | Hydrogenophaga | *Hydrogenophaga intermedia* | GCF_000723405.1 |
| Comamonadaceae | Hydrogenophaga | *Hydrogenophaga sp. Root209* | GCF_001428625.1 |
| Comamonadaceae | Hylemonella | *Hylemonella gracilis str. Niagara R* | GCF_000600295.1 |
| Comamonadaceae | Limnohabitans | *Limnohabitans sp. 103DPR2* | GCF_001412575.1 |
| Comamonadaceae | Ottowia | *Ottowia thiooxydans DSM 14619* | GCF_000422885.1 |
| Comamonadaceae | Pelomonas | *Pelomonas sp. Root662* | GCF_001427705.1 |
| Comamonadaceae | Polaromonas | *Polaromonas naphthalenivorans CJ2* | GCF_000015505.1 |
| Comamonadaceae | Pseudorhodoferax | *Pseudorhodoferax sp. Leaf267* | GCF_001422405.1 |
| Comamonadaceae | Pseudorhodoferax | *Pseudorhodoferax sp. Leaf274* | GCF_001422445.1 |
| Comamonadaceae | Ramlibacter | *Ramlibacter tataouinensis TTB310* | GCF_000215705.1 |
| Comamonadaceae | Ramlibacter | *Ramlibacter sp. Leaf400* | GCF_001424545.1 |
| Comamonadaceae | Rhodoferax | *Rhodoferax ferrireducens T118* | GCF_000013605.1 |
| Comamonadaceae | Rhodoferax | *Rhodoferax saidenbachensis ED16* | GCF_000498435.1 |
| Comamonadaceae | Roseateles | *Roseateles depolymerans* | GCF_001483865.1 |
| Comamonadaceae | Simplicispira | *Simplicispira psychrophila DSM 11588* | GCF_000688255.1 |
| Comamonadaceae | Variovorax | *Variovorax paradoxus S110* | GCF_000023345.1 |
| Comamonadaceae | Variovorax | *Variovorax paradoxus EPS* | GCF_000184745.1 |
| Comamonadaceae | Variovorax | *Variovorax paradoxus 110B* | GCF_000382045.1 |
| Comamonadaceae | Verminephrobacter | *Verminephrobacter eiseniae EF01-2* | GCF_000015565.1 |
| Comamonadaceae | Xenophilus | *Xenophilus azovorans DSM 13620* | GCF_000745855.1 |
| Enterobacteriaceae | Arsenophonus | *Arsenophonus endosymbiont str. Hangzhou of Nilaparvata lugens* | GCF_000757905.1 |
| Enterobacteriaceae | Buchnera | *Buchnera aphidicola str. Sg (Schizaphis graminum)* | GCF_000007365.1 |
| Enterobacteriaceae | Buchnera | *Buchnera aphidicola str. Bp (Baizongia pistaciae)* | GCF_000007725.1 |
| Enterobacteriaceae | Buchnera | *Buchnera aphidicola str. APS (Acyrthosiphon pisum)* | GCF_000009605.1 |
| Enterobacteriaceae | Buchnera | *Buchnera aphidicola BCc* | GCF_000090965.1 |
| Enterobacteriaceae | Buchnera | *Buchnera aphidicola (Cinara tujafilina)* | GCF_000217635.1 |
| Enterobacteriaceae | Buchnera | *Buchnera aphidicola str. Ak (Acyrthosiphon kondoi)* | GCF_000225445.1 |
| Enterobacteriaceae | Buchnera | *Buchnera aphidicola str. Ua (Uroleucon ambrosiae)* | GCF_000225465.1 |
| Enterobacteriaceae | Buchnera | *Buchnera aphidicola str. G002 (Myzus persicae)* | GCF_000521565.1 |
| Enterobacteriaceae | Budvicia | *Budvicia aquatica DSM 5075 = ATCC 35567* | GCF_000427805.1 |
| Enterobacteriaceae | Buttiauxella | *Buttiauxella agrestis* | GCF_000737905.1 |
| Enterobacteriaceae | Cedecea | *Cedecea neteri* | GCF_000757825.1 |
| Enterobacteriaceae | Citrobacter | *Citrobacter freundii CFNIH1* | GCF_000648515.1 |
| Enterobacteriaceae | Cronobacter | *Cronobacter sakazakii ATCC BAA-894* | GCF_000017665.1 |
| Enterobacteriaceae | Cronobacter | *Cronobacter zurichensis LMG 23730* | GCF_000463155.1 |
| Enterobacteriaceae | Dickeya | *Dickeya solani IPO 2222* | GCF_000400795.1 |
| Enterobacteriaceae | Edwardsiella | *Edwardsiella ictaluri 93-146* | GCF_000022885.2 |
| Enterobacteriaceae | Enterobacter | *Enterobacter cloacae subsp. cloacae ATCC 13047* | GCF_000025565.1 |
| Enterobacteriaceae | Enterobacter | *Enterobacter lignolyticus SCF1* | GCF_000164865.1 |
| Enterobacteriaceae | Enterobacter | *Enterobacter aerogenes KCTC 2190* | GCF_000215745.1 |
| Enterobacteriaceae | Erwinia | *Erwinia amylovora CFBP1430* | GCF_000091565.1 |
| Enterobacteriaceae | Dickeya | *Dickeya dadantii 3937* | GCF_000147055.1 |
| Enterobacteriaceae | Erwinia | *Erwinia billingiae Eb661* | GCF_000196615.1 |
| Enterobacteriaceae | Erwinia | *Erwinia toletana DAPP-PG 735* | GCF_000336255.1 |
| Enterobacteriaceae | Erwinia | *Erwinia tracheiphila PSU-1* | GCF_000404125.1 |
| Enterobacteriaceae | Erwinia | *Erwinia mallotivora* | GCF_000590885.1 |
| Enterobacteriaceae | Erwinia | *Erwinia oleae* | GCF_000770305.1 |
| Enterobacteriaceae | Escherichia | *Escherichia coli str. K-12 substr. MG1655* | GCF_000005845.2 |
| Enterobacteriaceae | Escherichia | *Escherichia coli O157:H7 str. Sakai* | GCF_000008865.1 |
| Enterobacteriaceae | Escherichia | *Escherichia coli UMN026* | GCF_000026325.1 |
| Enterobacteriaceae | Escherichia | *Escherichia coli IAI39* | GCF_000026345.1 |
| Enterobacteriaceae | Escherichia | *Escherichia coli O83:H1 str. NRG 857C* | GCF_000183345.1 |
| Enterobacteriaceae | Escherichia | *Escherichia hermannii NBRC 105704* | GCF_000248015.1 |
| Enterobacteriaceae | Escherichia | *Escherichia coli O104:H4 str. 2011C-3493* | GCF_000299455.1 |
| Enterobacteriaceae | Escherichia | *Escherichia vulneris NBRC 102420* | GCF_000759795.1 |
| Enterobacteriaceae | Franconibacter | *Franconibacter pulveris DSM 19144* | GCF_000621185.1 |
| Enterobacteriaceae | Hafnia | *Hafnia alvei FB1* | GCF_000597785.2 |
| Enterobacteriaceae | Kosakonia | *Kosakonia sacchari SP1* | GCF_000300455.2 |
| Enterobacteriaceae | Klebsiella | *Klebsiella pneumoniae subsp. pneumoniae HS11286* | GCF_000240185.1 |
| Enterobacteriaceae | Kluyvera | *Kluyvera ascorbata ATCC 33433* | GCF_000735365.1 |
| Enterobacteriaceae | Leclercia | *Leclercia adecarboxylata ATCC 23216 = NBRC 102595* | GCF_000735515.1 |
| Enterobacteriaceae | Leminorella | *Leminorella grimontii ATCC 33999 = DSM 5078* | GCF_000439085.1 |
| Enterobacteriaceae | Lonsdalea | *Lonsdalea quercina subsp. quercina* | GCF_000688655.1 |
| Enterobacteriaceae | Moellerella | *Moellerella wisconsensis ATCC 35017* | GCF_001294465.1 |
| Enterobacteriaceae | Morganella | *Morganella morganii subsp. morganii KT* | GCF_000286435.2 |
| Enterobacteriaceae | Pantoea | *Pantoea ananatis LMG 5342* | GCF_000283875.1 |
| Enterobacteriaceae | Pantoea | *Pantoea dispersa EGD-AAK13* | GCF_000465555.1 |
| Enterobacteriaceae | Pantoea | *Pantoea rwandensis* | GCF_000759475.1 |
| Enterobacteriaceae | Pectobacterium | *Pectobacterium atrosepticum SCRI1043* | GCF_000011605.1 |
| Enterobacteriaceae | Phaseolibacter | *Phaseolibacter flectens ATCC 12775* | GCF_000518745.1 |
| Enterobacteriaceae | Photorhabdus | *Photorhabdus luminescens subsp. laumondii TTO1* | GCF_000196155.1 |
| Enterobacteriaceae | Photorhabdus | *Photorhabdus temperata subsp. khanii NC19* | GCF_000517265.1 |
| Enterobacteriaceae | Plesiomonas | *Plesiomonas shigelloides 302-73* | GCF_000392595.1 |
| Enterobacteriaceae | Proteus | *Proteus mirabilis HI4320* | GCF_000069965.1 |
| Enterobacteriaceae | Providencia | *Providencia stuartii MRSN 2154* | GCF_000259175.1 |
| Enterobacteriaceae | Providencia | *Providencia rettgeri Dmel1* | GCF_000314835.2 |
| Enterobacteriaceae | Providencia | *Providencia burhodogranariea DSM 19968* | GCF_000314855.2 |
| Enterobacteriaceae | Rahnella | *Rahnella aquatilis CIP 78.65 = ATCC 33071* | GCF_000241955.1 |
| Enterobacteriaceae | Salmonella | *Salmonella enterica subsp. enterica serovar Typhimurium str. LT2* | GCF_000006945.1 |
| Enterobacteriaceae | Salmonella | *Salmonella enterica subsp. enterica serovar Typhi str. CT18* | GCF_000195995.1 |
| Enterobacteriaceae | Serratia | *Serratia proteamaculans 568* | GCF_000018085.1 |
| Enterobacteriaceae | Serratia | *Serratia symbiotica str. Tucson* | GCF_000186485.1 |
| Enterobacteriaceae | Serratia | *Serratia marcescens FGI94* | GCF_000330865.1 |
| Enterobacteriaceae | Serratia | *Serratia plymuthica S13* | GCF_000438825.1 |
| Enterobacteriaceae | Serratia | *Serratia fonticola AU-AP2C* | GCF_000477615.1 |
| Enterobacteriaceae | Serratia | *Serratia marcescens subsp. marcescens Db11* | GCF_000513215.1 |
| Enterobacteriaceae | Serratia | *Serratia multitudinisentens RB-25* | GCF_000520015.2 |
| Enterobacteriaceae | Shigella | *Shigella flexneri 2a str. 301* | GCF_000006925.2 |
| Enterobacteriaceae | Shigella | *Shigella dysenteriae Sd197* | GCF_000012005.1 |
| Enterobacteriaceae | Shimwellia | *Shimwellia blattae DSM 4481 = NBRC 105725* | GCF_000262305.1 |
| Enterobacteriaceae | Sodalis | *Sodalis glossinidius str. ''morsitans''* | GCF_000010085.1 |
| Enterobacteriaceae | Tatumella | *Tatumella morbirosei* | GCF_000757425.2 |
| Enterobacteriaceae | Wigglesworthia | *Wigglesworthia glossinidia endosymbiont of Glossina brevipalpis* | GCF_000008885.1 |
| Enterobacteriaceae | Wigglesworthia | *Wigglesworthia glossinidia endosymbiont of Glossina morsitans morsitans (Yale colony)* | GCF_000247565.1 |
| Enterobacteriaceae | Xenorhabdus | *Xenorhabdus bovienii SS-2004* | GCF_000027225.1 |
| Enterobacteriaceae | Xenorhabdus | *Xenorhabdus nematophila ATCC 19061* | GCF_000252955.1 |
| Enterobacteriaceae | Xenorhabdus | *Xenorhabdus cabanillasii JM26* | GCF_000531755.1 |
| Enterobacteriaceae | Yersinia | *Yersinia pestis CO92* | GCF_000009065.1 |
| Enterobacteriaceae | Yersinia | *Yersinia enterocolitica subsp. enterocolitica 8081* | GCF_000009345.1 |
| Enterobacteriaceae | Yersinia | *Yersinia ruckeri ATCC 29473* | GCF_000754815.1 |
| Enterobacteriaceae | Yokenella | *Yokenella regensburgei ATCC 43003* | GCF_000239335.1 |
| Oxalobacteraceae | Collimonas | *Collimonas fungivorans Ter331* | GCF_000221045.1 |
| Oxalobacteraceae | Duganella | *Duganella zoogloeoides ATCC 25935* | GCF_000383895.1 |
| Oxalobacteraceae | Herbaspirillum | *Herbaspirillum seropedicae SmR1* | GCF_000143225.1 |
| Oxalobacteraceae | Herbaspirillum | *Herbaspirillum lusitanum P6-12* | GCF_000256565.1 |
| Oxalobacteraceae | Herbaspirillum | *Herbaspirillum massiliense JC206* | GCF_000312045.1 |
| Oxalobacteraceae | Herminiimonas | *Herminiimonas arsenicoxydans* | GCF_000026125.1 |
| Oxalobacteraceae | Janthinobacterium | *Janthinobacterium lividum* | GCF_000632025.1 |
| Oxalobacteraceae | Janthinobacterium | *Janthinobacterium agaricidamnosum NBRC 102515 = DSM 9628* | GCF_000723165.1 |
| Oxalobacteraceae | Janthinobacterium | *Janthinobacterium sp. CG23_2* | GCF_001485665.1 |
| Oxalobacteraceae | Massilia | *Massilia timonae CCUG 45783* | GCF_000315425.1 |
| Oxalobacteraceae | Massilia | *Massilia niastensis DSM 21313* | GCF_000382345.1 |
| Oxalobacteraceae | Massilia | *Massilia alkalitolerans DSM 17462* | GCF_000427785.1 |
| Oxalobacteraceae | Massilia | *Massilia sp. WG5* | GCF_001412595.1 |
| Oxalobacteraceae | Massilia | *Massilia sp. Leaf139* | GCF_001424165.1 |
| Oxalobacteraceae | Massilia | *Massilia sp. Root418* | GCF_001425265.1 |
| Oxalobacteraceae | Massilia | *Massilia sp. Root133* | GCF_001426525.1 |
| Oxalobacteraceae | Oxalobacter | *Oxalobacter formigenes HOxBLS* | GCF_000158475.2 |
| Oxalobacteraceae | Oxalobacter | *Oxalobacter formigenes OXCC13* | GCF_000158495.1 |
| Pseudomonadaceae | Azotobacter | *Azotobacter vinelandii DJ* | GCF_000021045.1 |
| Pseudomonadaceae | Cellvibrio | *Cellvibrio japonicus Ueda107* | GCF_000019225.1 |
| Pseudomonadaceae | Cellvibrio | *Cellvibrio gilvus ATCC 13127* | GCF_000218545.1 |
| Pseudomonadaceae | Cellvibrio | *Cellvibrio mixtus subsp. mixtus J3-8* | GCF_000766945.1 |
| Pseudomonadaceae | Pseudomonas | *Pseudomonas aeruginosa PAO1* | GCF_000006765.1 |
| Pseudomonadaceae | Pseudomonas | *Pseudomonas putida KT2440* | GCF_000007565.1 |
| Pseudomonadaceae | Pseudomonas | *Pseudomonas syringae pv. tomato str. DC3000* | GCF_000007805.1 |
| Pseudomonadaceae | Pseudomonas | *Pseudomonas fluorescens SBW25* | GCF_000009225.2 |
| Pseudomonadaceae | Pseudomonas | *Pseudomonas syringae pv. syringae B728a* | GCF_000012245.1 |
| Pseudomonadaceae | Pseudomonas | *Pseudomonas protegens Pf-5* | GCF_000012265.1 |
| Pseudomonadaceae | Pseudomonas | *Pseudomonas stutzeri A1501* | GCF_000013785.1 |
| Pseudomonadaceae | Pseudomonas | *Pseudomonas mendocina ymp* | GCF_000016565.1 |
| Pseudomonadaceae | Pseudomonas | *Pseudomonas entomophila L48* | GCF_000026105.1 |
| Pseudomonadaceae | Pseudomonas | *Pseudomonas fulva 12-X* | GCF_000213805.1 |
| Pseudomonadaceae | Pseudomonas | *Pseudomonas fluorescens F113* | GCF_000237065.1 |
| Pseudomonadaceae | Pseudomonas | *Pseudomonas fragi B25* | GCF_000250615.1 |
| Pseudomonadaceae | Pseudomonas | *Pseudomonas fuscovaginae UPB0736* | GCF_000251185.1 |
| Pseudomonadaceae | Pseudomonas | *Pseudomonas stutzeri TS44* | GCF_000263395.1 |
| Pseudomonadaceae | Pseudomonas | *Pseudomonas stutzeri CCUG 29243* | GCF_000267545.1 |
| Pseudomonadaceae | Pseudomonas | *Pseudomonas luteola XLDN4-9* | GCF_000282775.1 |
| Pseudomonadaceae | Pseudomonas | *Pseudomonas denitrificans ATCC 13867* | GCF_000349845.1 |
| Pseudomonadaceae | Pseudomonas | *Pseudomonas thermotolerans DSM 14292* | GCF_000364625.1 |
| Pseudomonadaceae | Pseudomonas | *Pseudomonas pelagia CL-AP6* | GCF_000410875.1 |
| Pseudomonadaceae | Pseudomonas | *Pseudomonas putida MTCC 5279* | GCF_000411615.1 |
| Pseudomonadaceae | Pseudomonas | *Pseudomonas resinovorans NBRC 106553* | GCF_000412695.1 |
| Pseudomonadaceae | Pseudomonas | *Pseudomonas caeni DSM 24390* | GCF_000421765.1 |
| Pseudomonadaceae | Pseudomonas | *Pseudomonas azotifigens DSM 17556* | GCF_000425625.1 |
| Pseudomonadaceae | Pseudomonas | *Pseudomonas alcaligenes OT 69* | GCF_000455385.1 |
| Pseudomonadaceae | Pseudomonas | *Pseudomonas alcaligenes NBRC 14159* | GCF_000467105.1 |
| Pseudomonadaceae | Pseudomonas | *Pseudomonas taeanensis MS-3* | GCF_000498575.1 |
| Pseudomonadaceae | Pseudomonas | *Pseudomonas oleovorans MOIL14HWK12* | GCF_000510765.1 |
| Pseudomonadaceae | Pseudomonas | *Pseudomonas stutzeri* | GCF_000590475.1 |
| Pseudomonadaceae | Pseudomonas | *Pseudomonas knackmussii B13* | GCF_000689415.1 |
| Pseudomonadaceae | Pseudomonas | *Pseudomonas japonica NBRC 103040 = DSM 22348* | GCF_000730585.1 |
| Pseudomonadaceae | Pseudomonas | *Pseudomonas alkylphenolia* | GCF_000746525.1 |
| Pseudomonadaceae | Pseudomonas | *Pseudomonas lutea* | GCF_000759445.1 |
| Pseudomonadaceae | Pseudomonas | *Pseudomonas rhizosphaerae* | GCF_000761155.1 |
| Pseudomonadaceae | Pseudomonas | *Pseudomonas sp. TTU2014-080ASC* | GCF_001446935.1 |
| Pseudomonadaceae | Rhizobacter | *Rhizobacter sp. Root29* | GCF_001424785.1 |
| Pseudomonadaceae | Rhizobacter | *Rhizobacter sp. Root1221* | GCF_001425785.1 |
| Pseudomonadaceae | Rhizobacter | *Rhizobacter sp. Root404* | GCF_001425865.1 |
