# Supplementary material for: Potential Mechanism of Detoxification of Cyanide Compounds by Gut Microbiomes of Bamboo-Eating Pandas
Source: mSphere. 2018 Jun 13;3(3):e00229-18. doi: 10.1128/mSphere.00229-18 (PMC6001608; doi:10.1128/mSphere.00229-18)
Supplement: TABLE S3 [file sph003182564st3.docx]

| **Family** | **Number of Genus** | **Number of Species** | **glpE** | **sseA** | **nit** |
| --- | --- | --- | --- | --- | --- |
| *Pseudomonadaceae* | 4 | 41 | 35 | 36 | 14 |
| *Clostridiaceae* | 12 | 88 | 0 | 2 | 26 |
| *Comamonadaceae* | 21 | 39 | 0 | 39 | 54 |
| *Enterobacteriaceae* | 40 | 81 | 70 | 63 | 29 |
| *Oxalobacteraceae* | 7 | 18 | 0 | 16 | 20 |

glpE, rhodanese /thiosulfate sulfurtransferases [EC2.8.1.1); sseA, 3-mercaptopyruvate sulfurtransferase; nit, Nitrilase [EC 3.5.5.1]
