# Supplementary material for: Potential Mechanism of Detoxification of Cyanide Compounds by Gut Microbiomes of Bamboo-Eating Pandas
Source: mSphere. 2018 Jun 13;3(3):e00229-18. doi: 10.1128/mSphere.00229-18 (PMC6001608; doi:10.1128/mSphere.00229-18)
Supplement: Supplementary file 4 [file sph003182564sf1.docx]

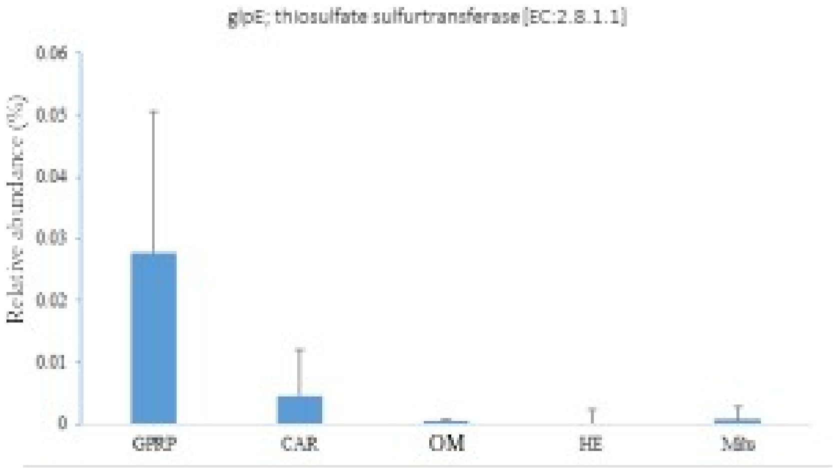
glpE; thiosulfate sulfurtransferase [EC:2.8.1.1]
Relative abundance (%)
0.06
0.05
0.04
0.03
0.02
0.01
0
GPRP
CAR
OM
HE
Mix
