# Supplementary material for: Potential Mechanism of Detoxification of Cyanide Compounds by Gut Microbiomes of Bamboo-Eating Pandas
Source: mSphere. 2018 Jun 13;3(3):e00229-18. doi: 10.1128/mSphere.00229-18 (PMC6001608; doi:10.1128/mSphere.00229-18)
Supplement: Supplementary file 5 [file sph003182564sf2.docx]

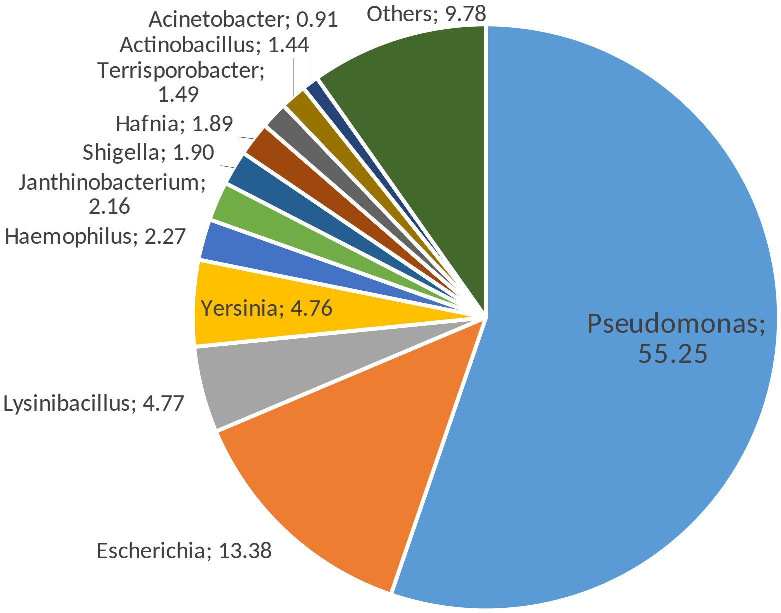
Acinetobacter; 0.91
Actinobacillus; 1.44
Terrisporobacter; 1.49
Hafnia; 1.89
Shigella; 1.90
Janthinobacterium; 2.16
Haemophilus; 2.27
Yersinia; 4.76
Lysinibacillus; 4.77
Escherichia; 13.38
Others; 9.78
Pseudomonas; 55.25
