# Supplementary material for: Potential Mechanism of Detoxification of Cyanide Compounds by Gut Microbiomes of Bamboo-Eating Pandas
Source: mSphere. 2018 Jun 13;3(3):e00229-18. doi: 10.1128/mSphere.00229-18 (PMC6001608; doi:10.1128/mSphere.00229-18)
Supplement: Supplementary file 6 [file sph003182564sf3.docx]

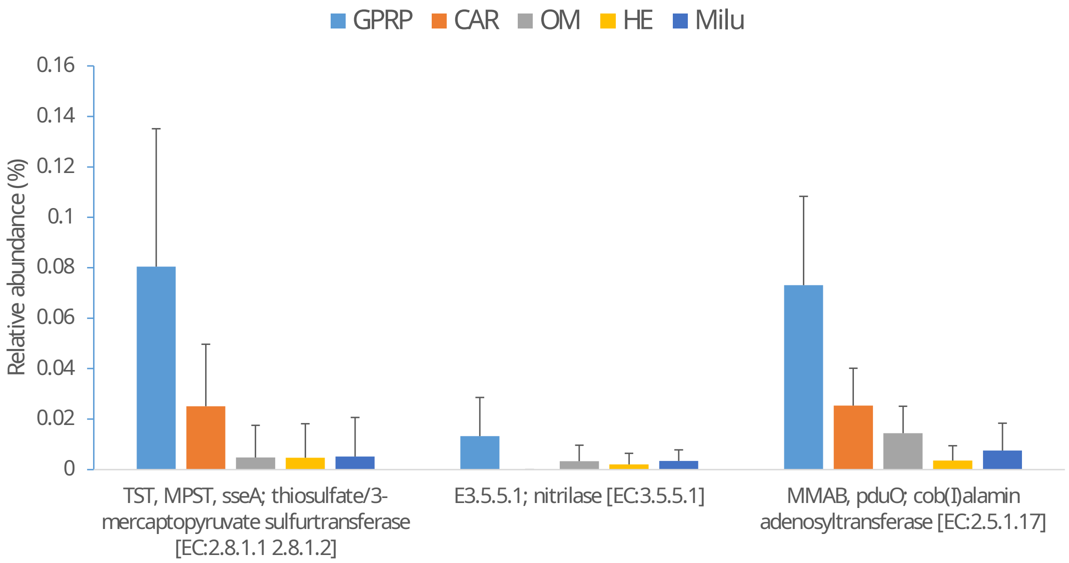

GPRP
CAR
OM
HE
Milu
Relative abundance (%)
0.16
0.14
0.12
0.1
0.08
0.06
0.04
0.02
0
TST, MPST, sseA; thiosulfate/3-mercaptopyruvate sulfurtransferase [EC:2.8.1.1 2.8.1.2]
E3.5.5.1; nitrilase [EC:3.5.5.1]
MMAB, pduO; cob(I)alamin adenosyltransferase [EC:2.5.1.17]
