# Supplementary material for: Potential Mechanism of Detoxification of Cyanide Compounds by Gut Microbiomes of Bamboo-Eating Pandas
Source: mSphere. 2018 Jun 13;3(3):e00229-18. doi: 10.1128/mSphere.00229-18 (PMC6001608; doi:10.1128/mSphere.00229-18)
Supplement: Supplementary file 7 [file sph003182564sf4.docx]

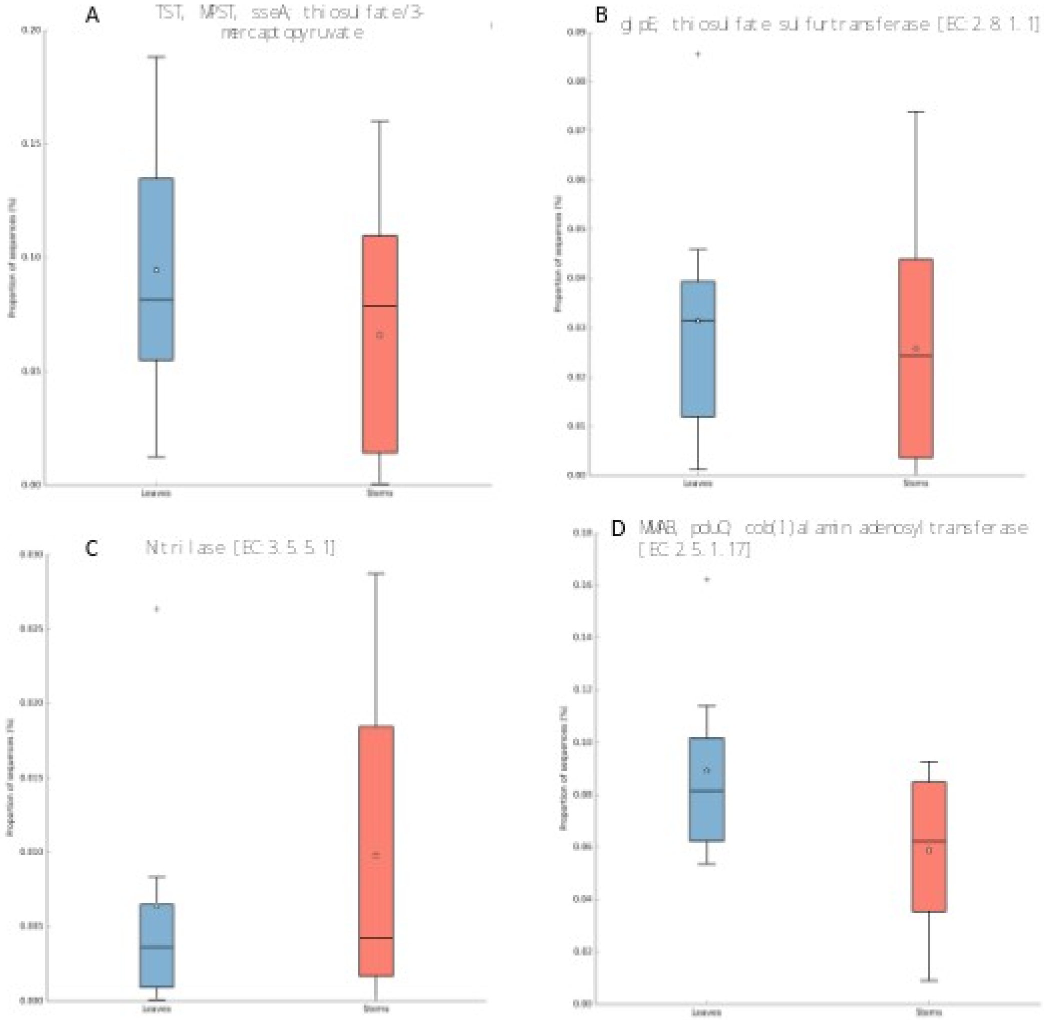
A
TST, MPST, sseA; thiosulfate/3-mercaptopyruvate
B
glpE; thiosulfate sulfurtransferase [EC:2.8.1.1]
C
Nitrilase [EC:3.5.5.1]
D
MMAB, pduO; cob(I)alamin adenosyltransferase [EC:2.5.1.17]
Proportion of sequences (%)
Leaves
Stems
